# Combination of Cell-Penetrating Peptides with Nanoparticles for Therapeutic Application: A Review

**DOI:** 10.3390/biom9010022

**Published:** 2019-01-10

**Authors:** Sara Silva, António J. Almeida, Nuno Vale

**Affiliations:** 1 Laboratory of Pharmacology, Department of Drug Sciences, Faculty of Pharmacy, University of Porto, Rua de Jorge Viterbo Ferreira 228, 4050-313 Porto, Portugal; nuno.vale@ff.up.pt; 2 Research Institute for Medicines (iMed.ULisboa), Faculty of Pharmacy, Universidade de Lisboa, 1964-003 Lisbon, Portugal; aalmeida@ff.ulisboa.pt; 3 Institute of Molecular Pathology and Immunology of the University of Porto (IPATIMUP), Rua Júlio Amaral de Carvalho, 45, 4200-135 Porto, Portugal; 4 Instituto de Investigação e Inovação em Saúde (i3S), University of Porto, Rua Alfredo Allen, 208, 4200-135 Porto, Portugal; 5 Department of Molecular Pathology and Immunology, Institute of Biomedical Sciences Abel Salazar (ICBAS), University of Porto, Rua de Jorge Viterbo Ferreira 228, 4050-313 Porto, Portugal

**Keywords:** cell-penetrating peptides, membrane translocation, delivery systems, therapeutic molecule, nanoparticles

## Abstract

Cell-penetrating peptides (CPPs), also known as protein translocation domains, membrane translocating sequences or Trojan peptides, are small molecules of 6 to 30 amino acid residues capable of penetrating biological barriers and cellular membranes. Furthermore, CPP have become an alternative strategy to overcome some of the current drug limitations and combat resistant strains since CPPs are capable of delivering different therapeutic molecules against a wide range of diseases. In this review, we address the recent conjugation of CPPs with nanoparticles, which constitutes a new class of delivery vectors with high pharmaceutical potential in a variety of diseases.

## 1. Cell-Penetrating Peptides

The limited ability of current drugs to achieve penetration of the interior of the cell has contributed to their lack of biological action, decreased therapeutic efficacy and enhanced toxicity. These limitations have potentiated the development of alternative strategies, routes, vectors of delivery and administration methods. Over the last years, different cellular translocation techniques have been developed including physical methods—electroporation and microinjection; chemical methods—calcium phosphate; and viral vectors. However, these methods have been considered highly toxic to cells, thus, decreasing the cellular viability and potentiating viral recombination in the case of viral vectors [1]. Ongoing research has discovered some protein transduction domains of peptides that are capable of translocating cell membrane efficiently without compromising their integrity [2]. These are cell-penetrating peptides (CPPs), normally with a maximum length of 30 amino acid residues, and are characterized by a high content of basic amino acid residues, resulting in an overall positive net charge. CPPs are advantageous over other translocation methods because they possess high cellular permeability rates, ability to translocate into a wide spectrum of cell types, large cargo capacity and low cell toxicity associated with no immunological response [3]. 

The first CPP, discovered in 1988, by Frankel et al. was HIV-Tat protein responsible for virus replication and considered a powerful transactivation factor of viral and cellular gene expression [4]. HIV-Tat of 86 amino acid residues act as a viral growth factor and is taken up by the cells [4]. Some years later, Vives et al. recognized the domain responsible for the rapid penetration of this peptide through plasma membrane—the truncated version of the HIV-Tat basic domain (48–57) [5]. In addition, *Drosophila antennapedia* homeobox sequence (pAntp) was reported as capable of entering nerve cells and accumulating in the nucleus [6]. In 1994, Derossi et al. identified Penetratin, a 16-mer peptide derived from the C-terminal region of the third helix of the homeodomain of Antennapedia and capable of penetrating cells [7,8]. The discovery of these two peptides with penetrating activities prompted more studies and the discovery of new CPPs. Over the next years, more CPPs were added to the list and nowadays a database has been built that permits one to browse existing CPPs based on chemical modifications, category, cargo or peptide length [9] (Table 1). In this review we will address CPPs mechanisms of penetration and the recent papers on the conjugation of CPPs with complex structures such as nanoparticles (NPs) [10,11,12], micelles [13] or liposomes [14], which results in enhanced stability, bioavailability and efficiency of CPPs.

### 1.1. Mechanism of Penetration

The exact mechanism of CPPs cell penetration still remains unknown [28]. Over the years, some discrepancies between studies have generated controversial data. A variety of features and conditions are crucial to the efficiency of cellular translocation and mode of penetration, and influence the different outcomes observed, such as cargo, cell type, incubation time, sample handling (washing, fixation, delays), fluorophore, CPPs concentration, cell density, stage of cell cycle, read out (uptake distribution), etc. [29,30,31]. One of the first problems reported was the over-quantification of peptide uptake, and respective localization in the interior of the cell. The experimental fixation procedure, even when mild conditions were applied, lead to wrong statements about direct translocation for all the peptides [32]. The cell sort flow cytometry presented another problem with overestimated uptake, since, at the time it was not possible to differentiate between membrane bound peptides and peptides in the interior of the cell without previous treatment with trypsin [32,33]. Before 2003, direct penetration was considered the main route for CPPs penetration, until some authors assumed a possible misleading error found in the fixation protocol when comparing the results with those observed for living cells, which questioned the possibility of the existence of other types of endocytic mechanisms inherent to CPPs [33], and demanded an extra revaluation of the mechanism of CPPs penetration. Nowadays, many authors have investigated the mechanism of cell uptake and it has become clear that different pathways can be used by CPPs depending on the conditions [34], and different effects on cellular membranes have been observed, such as curvature changes, alterations in membrane domain architecture, non-bilayer disorder, fusion of vesicles and/or lipid flip-flop processes [35]. The different pathways can be divided into two groups: energy-independent direct penetration and energy-dependent endocytosis [36]. 

The process of direct penetration can be tested with the use of specific experimental conditions such as low temperature, ATP depletion and in the presence of endocytic inhibitors. The first step of uptake begins with initial electrostatic interactions between positively charged CPP and negatively charged membrane components such as the phospholipid bilayer, leading to the CPP entrance. For instances, CPP can penetrate cellular membrane through different model mechanisms such as transient pore formation or membrane destabilization [37,38]. 

The transient pore formation models are characterized by the formation of toroidal pores or barrel-stave pores on the cellular membrane. The barrel-stave model, described by Gazit and et al. in 1994, is characterized by an initial interaction of CPP with cell membrane, which assume an amphipathic α-helix structure as the basic residues interact [39]. Then, CPP inserts in the membrane and cluster together in a barrel-like structure in such a way that the hydrophobic region interacts with the lipid core and the hydrophilic region faces the interior of the transient pore [39]. 

The other two models responsible for membrane destabilization are the carpet-model and inverted micelle model. In 1992, Pouny et al. proposed the carpet-model, which stipulates that positively charged amphipathic CPPs bind to acid phospholipids head groups and dispose horizontally through the membrane surface in a “carpet-like” fashion [37]. Further interactions of the hydrophobic region with the polar region of the membrane and positive charges directed towards the hydrophilic region consequently leads to destabilization of the cellular membrane and internalization of CPPs [37]. The inverted micelle model was first described with pAntp CPP. Derossi et al. proposed that positively charged peptides interact with membranes dislocating negatively charge phospholipids to the site, inducing the formation of an inverted micelle and release of CPPs into the intracellular compartment [8].

Even though direct penetration was initially proposed as the main mechanism of CPPs internalization, later, in 2003, biophysical studies elucidated in detail the mechanisms of permeation of lipid bilayer and suggested that endocytosis could also represent the main entry route for many CPPs [30]. Endocytosis comprises two distinct mechanisms of cell entry subdivided in two groups: (1) phagocytosis of large molecules are usually restricted to specific types of cells responsible for the elimination of pathogens such as macrophages, monocytes and neutrophils; and (2) pinocytosis occurs in all cells. Focusing on pinocytosis, there are four different pathways: macropinocytosis, clatherin endocytosis, caveolae/lipid raft mediated endocytosis and clatherin and caveolae independent endocytosis, which differ on the size of endosome, nature of the cargo and mechanism of vesicle formation [34,40]. In order to study which mechanism is associated with CPPs entrance and escape from endosome to cytoplasm different experimental techniques and strategies can be applied. 

To understand the specific mechanisms and involvement of certain proteins such as clatherin and caveolin, the experimental procedures that have been adopted used specific inhibitors such as amiloride (inhibits macropinocytosis) Dynasore, chlorpromazine and sucrose (clatherin dependent endocytosis), nystactin (caveolae-mediated) depletion of cholesterol (inhibits lipid raft endocytosis) and more [22,38,41,42,43,44]. 

Nonetheless, after endocytosis, CPPs or CPP-cargo conjugated are confined in endosomes and risk exposure to degradation from lysosomes. Thus, escaping from endosomes is a crucial step in order for them to reach their target site and execute their biological activity. The mechanism of endosomal escape is still poorly understood. However, some hypotheses have been presented. One hypothesis of endosomal escape is that it is determined by pH gradient, with vesicle maturation the pH decreases and promotes increased CPP interactions with membranes, and consequently, their release occurs [32]. Another model proposes that positively charged CPPs, once inside the vesicle, interacts with negatively charged components of the endosomal membrane, which contributes to tightening the membrane, determines its disruption and the release of CPPs [45]. A variety of agents have been designed to promote endosomal escape such as the lysosomotropic agent chloroquine (CQ) [43], a classical antimalarial drug. Another approach is the introduction of histidine moieties in the CPP sequence, which have a “proton sponge effect” and increase the osmotic pressure inside the endosome [46,47]. Similar to this concept is the insertion of GALA, KLA and KALA sequences in the CPP sequence resulting in endosomal escape [11]. A different strategy fused CPP with influenza virus sequence (dTat-HA2) and this enhanced macropinocytic escape [41,48]. The work of Oba et al. added an unnatural amino acid (lysine derivative) with a guanidinylethyl group in the side chain of amine to the arginine-rich peptide and the degree of protonation in the guanidinylethyl amine structure played a crucial role in endosome escape abilities [46].

### 1.2. Cell-Penetrating Peptide Therapeutics Potential: Delivery Molecules

Since the discovery of the potential therapeutic approach of CPPs as safe and efficient delivery vectors, an increased number of studies have examined CPP-cargo transfection and modulation of cells and tissues both in vitro and in vivo. Different molecules such as peptides, proteins, drugs, antisense oligonucleotides, dyes, siRNAs have already been conjugated to CPPs and used in both research and therapeutic applications [1,47]. The CPP-cargo conjugation has not only been widely studied but has also demonstrated interesting results so far in targeting different pathologies such as inflammation, ischemia, cardiovascular disease, psoriasis [49], their use as imaging agents [50,51,52], cancer and neurodegenerative diseases [53,54]. Besides its therapeutic potential in oncologic conditions and neurologic disorders, the research focus is also on clarifying how CPPs can cross resistant tumour cells and be highly selective to the almost impermeable blood-brain barrier (BBB) [53,54]. Another recent approach involving CPPs, is the possibility of delivering biopharmaceuticals through a non-invasive route. Some biopharmaceuticals are still administered through parental injection, which is associated with discomfort, pain and low patient compliance. The ideal administration route must be well tolerated and accepted among patients, preferably in oral dosage forms. Patients with chronic diseases require frequent injections and transition to oral forms of administration would increase the quality of life for millions of people. Nevertheless, successful oral delivery is still a big challenge, in part due to the inherent disadvantages of the gastro-intestinal tract that presents inter-individual and intra-individual variations, physical and biochemical barriers of intestinal mucosa, pH alterations and exposure to enzyme degradation. Potential oral use of CPPs has been presented in several research papers in order to facilitate and improve permeation of drugs and therapeutic proteins across intestinal epithelium [55]. For this purpose, scientists have implemented different strategies for gene-targeted therapy with regulation of gene expression [50,56].

Although CPPs have been presented as useful and remarkable delivery tools, there are still some challenges to be addressed. Some of the barriers are associated with in vivo results such as poor bioavailability, low stability, lack of selectivity, reduced accuracy and short lifespan [31]. The CPP incorporation of L-enantiomers instead of D-amino acid analogues and/or of unnatural amino acid enhances the stability and bioavailability of peptides in serum [57,58]. Another successful approach is the cyclization of CPP structure [59]. The enhancement of efficiency with cyclization of arginine-rich peptides has been demonstrated. The authors found maximal separation of guanidium groups by cyclization of the peptide structure and enhanced their non-endocytic cellular uptake [60]. The work of Gronewold et al. demonstrated that dimerization of CPP sC18 promoted increased cellular uptake (mainly direct penetration) and tumour cell selectivity, depending on the composition of target membrane (MCF-7 cancer cells) [61]. Another important topic is the CPP’s lack of selectivity, which can be addressed by the incorporation of sequences of targeting [54,62,63] to specific receptors [53] and the use of mRNA display technology to design tumour lineage-homing peptides [22]. Furthermore, incorporation or conjugation of CPPs with complex structures such as NP [10,64], micelles [13] or liposomes [14] promotes stability, bioavailability and in vivo efficiency of CPPs.

## 2. Nanoparticles

**Figure 1:**
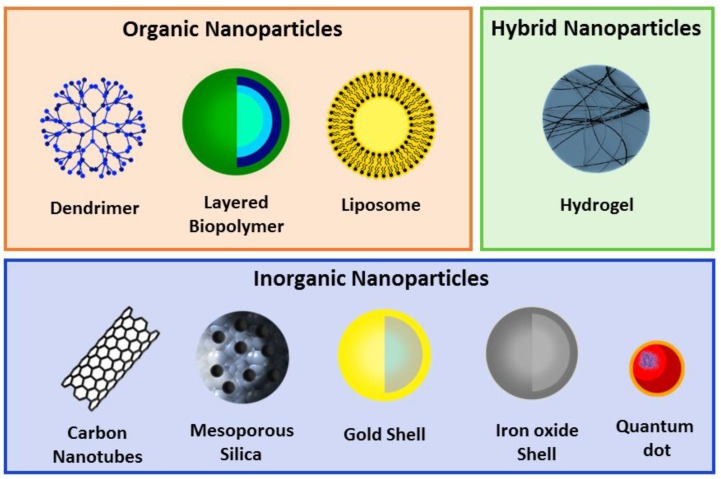
Schematic representation of different types of nanoparticles (NPs) divided into organic, hybrid and inorganic categories.

The possibility of controlling drug delivery promoted the development of the “PEGlylaton concept” by Frank Davis who demonstrated that molecules with poly(ethylene glycol) (PEG) presented reduced immunogenicity [65] and could increased CPPs circularization time. Later, in 1965, Feynman introduced the nanotechnology concept with the discovery of the potentially therapeutic capabilities of liposomes in drug delivery [66]. In 1970, Langer explored the abilities of non-degradable polymer matrices (polynyinilacetate) to deliver and release proteins [67]. Through the years, several advances have been made in the field of nanostructures delivery systems. The definition of a NP is: *“Nanoparticles may be defined as being submicronic (<1 uM) colloidal systems generally but not necessarily made of polymers”* [68]. Nanoparticles can be divided into three groups: organic (lipids, proteins or polymers); hybrid (nanofoams); and inorganic (metals or salts); and present a variety of structural forms such as polymeric nanoconstructs, nanomembranes, nanotubes, nanofibers and nanosized silicon drips (Figure 1) [69]. Nanoparticles are able to mimic or alter biological processes and have been widely studied as potential delivery vectors to treat cancer, cardiovascular diseases, central nervous system (CNS) disorders, infection diseases, imaging probes and more [66].

Nanotechnology-based alternatives were developed to address the current limitations of drugs. The field of NP-pharmacokinetics is the main area for obtaining maximal potential with efficient absorption and the right biodistribution [66]. For that, NP optimization with surface modifications is required to design a more effective and successful therapeutic. Different possible modifications have been investigated for passive or active drug targeting (this subject is excellently reviewed for lipid NP by Gaspar et al. (2017) [70]). For example, coating NP with hydrophobic molecules, such as PEG, polyoxamer, poloxamine and polysorbate 80 (Tween 80), is associated with prolonged circulation time and reduced phagocytosis.

In addition, NP can be functionalized with peptides (CPPs, avidin-biotin), antibodies, transferrins and saccharides (mannose, hyaluronic acid) in order to achieve specific interactions between the target ligand and NP conjugates [70].

For instance, scientists used photon-correlation spectroscopy and dynamic light scattering to determine NP size, which is crucial to understanding theNP’s biological fate, toxicity, drug release and stability [69]. Another crucial analysis required is the surface properties of NP, such as hydrophobicity that influence their in vivo fate and the zeta potential. The zeta potential (surface charge) reflects the electric potential of theNP, which is influenced by the composition and surrounding medium. Zeta potential above ±30 mV shows NPstability in suspensions, reduced aggregation and can be used to determine where active material is encapsulated [61].

Nevertheless, several limitations have delayed NPusage in current therapeutics, mostly because of poor drug loading, rapid release and lack of selectivity [71]. The current limitations of NPhave boosted the design of new alternatives conjugates.

## 3. Cell-Penetrating Peptide Conjugation with Nanoparticles for Therapeutic Applications

So far intracellular delivery of therapeutic molecules has been one of the major challenges to achieving successful treatment. The biological cell membrane acts as a barrier to proteins and specific compounds unless an active transport mechanism is involved. Certain pharmaceutical nanocarriers have been designed to facilitate the drug delivery inside cells, and increase the stability of the administered drug. Among all delivery vectors, CPPsand nanoparticles present the greatest potential, however, some limitations are still associated with this.

**Figure 2:**
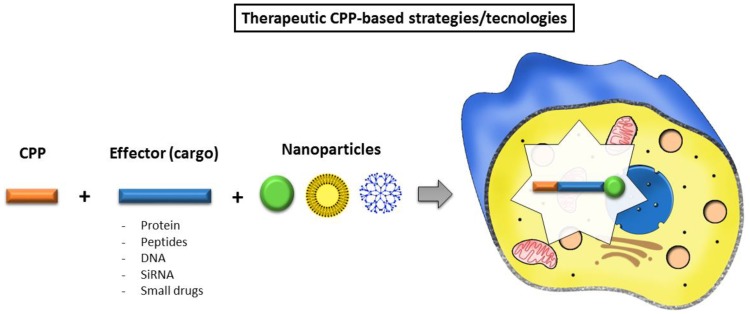
Schematic representation of recent CPP-based strategies. This dual conjugation of cell-penetrating peptide (CPP), nanoparticle and effector (cargo) can further potentiate the therapeutic effectiveness and enhance stability.

The conjugation of CPPs with NPhas been analyzed in order to fulfil the gap between both molecules, and to potentiate the development of a new compound/conjugate that possesses enhanced efficiency, accuracy and therapeutic activity (Figure 2; Table 2). Many studies have demonstrated successful results in this conjugation to treat variety of diseases, such as dermatological disorders (topical application) [72], cancer [64,73], as imaging probes, inflammation [74] and CNS disorders; some of these will be addressed in this review (see Table 3).

### 3.1. Cell-Penetrating Peptides and Nanoparticles in Cancer

**Figure 3:**
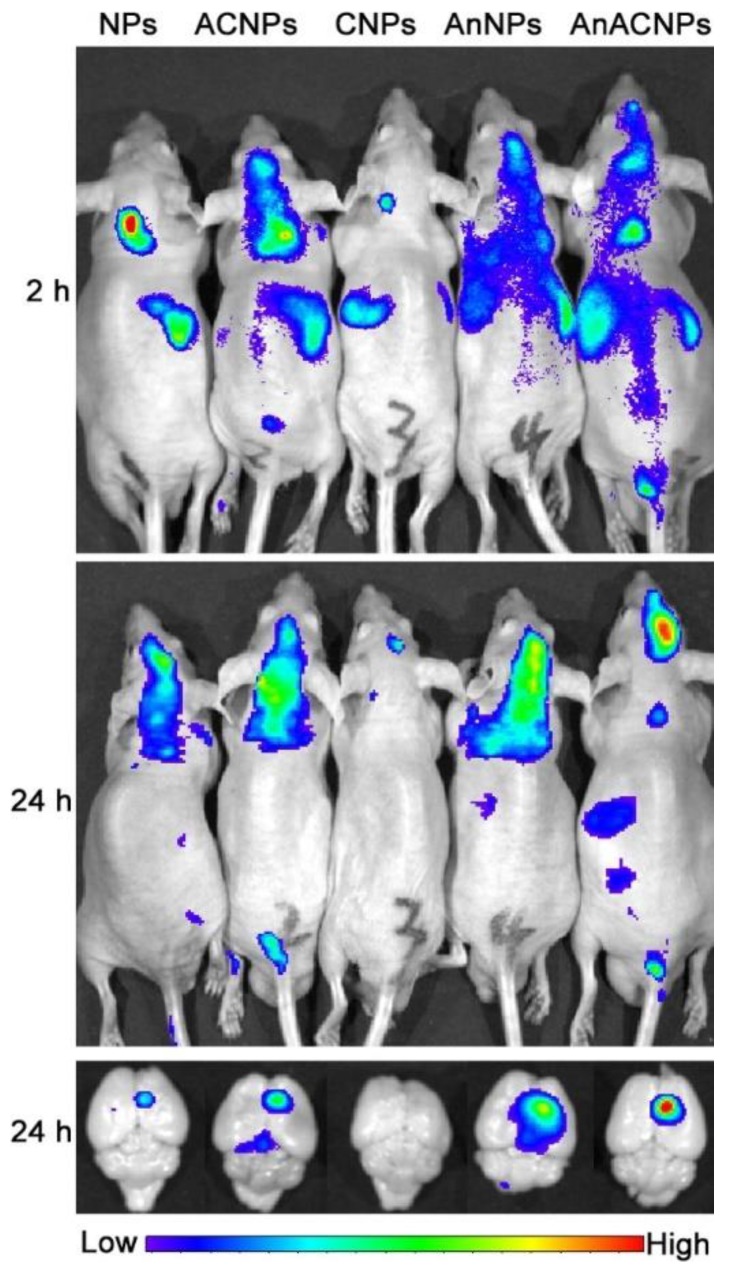
In vivo imaging of whole body and ex vivo imaging of brain from mice that were treated with different DiR-loaded formulations. Adapted from Molecular Pharmaceutics with permission from the American Chemical Society, published by American Chemical Society 2014, Copyright 2014 [81].

The treatment of cancer with chemotherapy drugs lacks specificity and often results in toxicity and many side-effects for patients. Conventional chemotherapy shows poor water solubility, low tumour specificity, high toxicity and rapid blood clearance. Another limitation that can be added to the list is the emergence of multi-drug resistance, making cancer one of the most concerning health problems, and life threatening diseases worldwide [75]. Cancer is characterized as a complex disease with tumour heterogeneity between cancers and among individuals. In order to achieve more successful treatment and reduce side effects, chemotherapy has been conjugated with NP as delivery systems [76]. However, limitations continue to persist and tumour penetration is required. Therefore, new strategies and the addition of new moieties can enhance anti-tumour efficacy and promote tumour penetration. The CPP/NP-cargo delivery can enable targeted treatment and eradication of tumours without affecting healthy tissues. Several studies have exploited this CPP/NP potential as delivery systems in cancer cell-lines and cancer animal models. One of the most challenging barriers to cross is the BBB, which is crucial for maintenance of brain homeostasis and protection against organisms and toxins and translocation of molecules is limited. The work of Li et al. demonstrated the potential of a 12-mer peptide that targets the brain—TGN. Conjugation of TGN with PEG-poly(lactic-co-glycolic acid) (PEG-PGLA) NPwere able to deliver coumarin-6 as a probe into the brain at 3.6-fold times higher than NP alone [16]. Using the same brain peptide sequence, Gao et al. analyzed the potential of TGN peptide and A5411 aptamer conjugated with PEG-poly(ε-caprolactone) (PEG-PCL) NP to deliver docetaxel (DTX) in brain glioma bearing mice [77]. Aptamers are short sequences of RNA or DNA that enables the complex to bind to a specific protein. In this work, aptamers A5411 was able to bind necleotin, which is highly expressed protein in plasma membrane cancer promoting dual selectivity with TGN peptide. The authors demonstrated the success of the treatment, which was reflected in increased survival rates in treated mice and improvements in anti-tumour activity compared to saline and drug-free treated mice [77]. Glioblastoma multiform is a primary central nervous system tumour and the second cause of death in patients less than 35 years old. The pathogenesis of the tumour is characterized by an increase diffusion rate, which makes it almost impossible to remove completely with surgery, and chemotherapy cannot reach the tumour at the desirable concentration [78]. Therefore, Xin et al. studied the potential of CPP/NP conjugation to target CNS multiform glioblastoma tumours [79]. In this work, the authors engineered a new multifunctional carrier with CPP Angiopep-2 and NPPEG-PCL loaded with placlitaxel (PTX). The CPP angiopep-2 targets BBB and glioma tumour cell because it binds to the low-density lipoprotein receptor-related protein (LRP) that is highly expressed in human glioma cell and BBB. The authors demonstrated that the dual-targeting drug delivery system promoted inhibition and apoptosis against U87M6 glioma cells in a monolayer model, and accumulated in the glioma site in vivo [79]. Due to the target selectivity of Angiopep-2, this sequence was further used in other constructs involving glioblastoma targeted delivery. The work of Mei et al. demonstrated the potential of this Angiopep-2 combined with NP and the addition of an activable CPP (ACPP) in the construct [80]. The ACPP takes advantage of target site microenvironment differences such as pH, redox potential, and up regulated protein to turn inactive molecules into active ones [12]. The stimulus-sensitive drug delivery system is able to specifically target tumours. Mei’s work used an ACPP with PLGGLAG sequence which is the subtract of metalloproteinase 2 (MMP2) that is over expressed in tumour cells. The authors demonstrated that dual functionalization further improves the tumour target efficiency in vivo [80]. In another interesting publication, this construct was further investigated to deliver DTX as a drug model to evaluate glioma-targeting ability and treatment efficiency. The authors demonstrated through ex vivo imaging (Figure 3) the target selectivity of the dual construct to the brain and into the tumour site after 24 h. In addition, the conjugate angiopep-2 and ACPP dual-modified NPs (AnACNPs) showed a high anti-tumour effect, reflected by higher survival rates in treated mice over the control [81].

**Figure 4:**
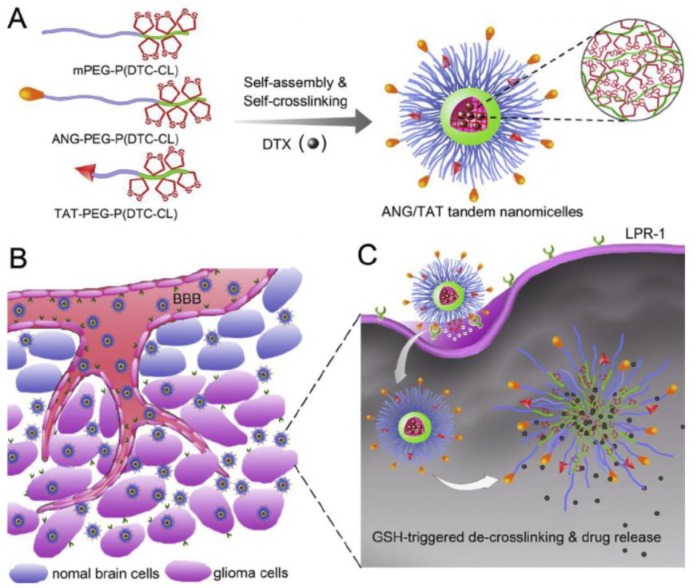
Illustration of tandem nanomicelles co-functionalized with brain tumor-targeting and CPPs (Angiopep-2 and TAT) for specific anti-glioma chemotherapy. (**A**) ANG/TAT-Ms micelles are assembled from PEG; (**B**) Tandem nanomicelles are able to cross blood-brain barrier (BBB) and target glioma cells; (**C**) Docetaxel (DTX) is released into the cytoplasm as a result of Glutathione (GSH)-triggered de-cross-linking of ANG/TAT-Ms. Adapted from Journal of Controlled Release with permission from correspondent’s authors Zhang, J., Feijen J. and Zhong Z., published by Elsevier 2018, Copyright 2018 [82].

The dual-functionalization of NP was further tested in the work of Zhu et al., which designed crosslinks of tandem nanomicelles with both Angiopep-2 and a TAT shield with PEG6000 for anti-glioma chemotherapy. In vivo results with the treatment with DTX/ANG20/TAT10-MS demonstrated inhibition of tumour growth and longest survival time of 53 days compared to 29 days for free DTX (Figure 4) [82].

**Figure 5:**
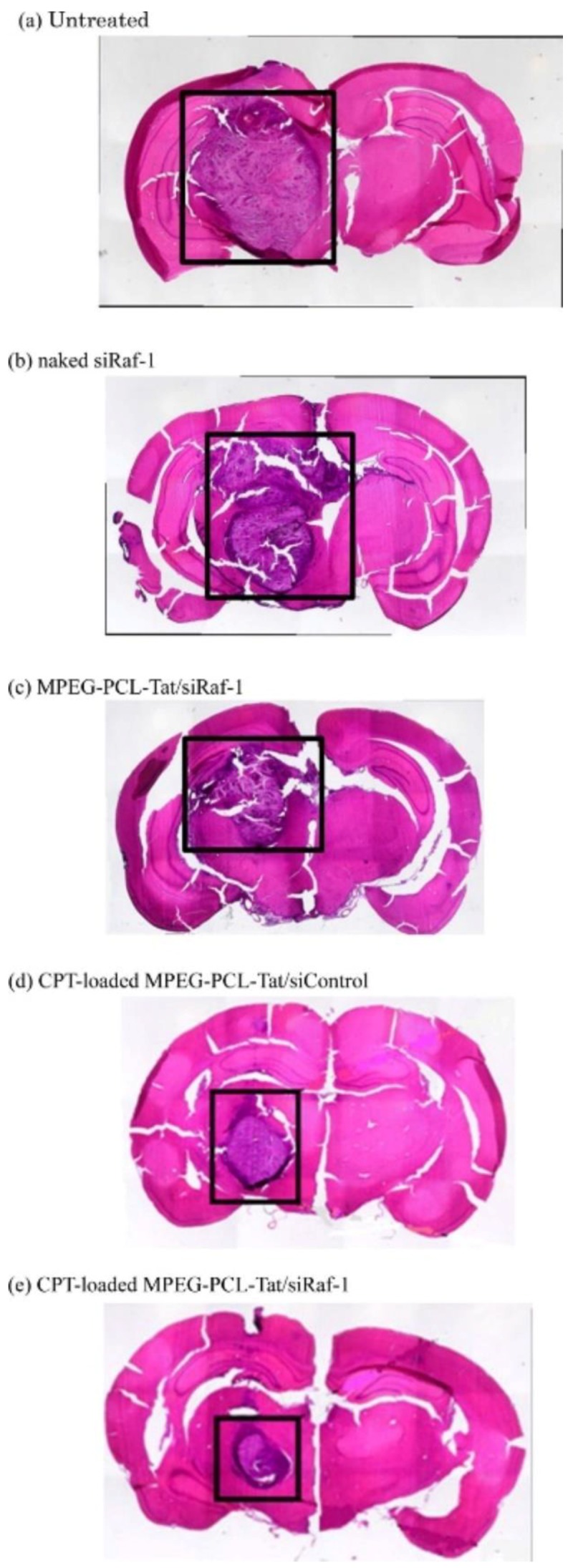
Photographs of HE-stained brain tissue in intracranial C6 glioma-bearing rats after intranasal administration of siRaf-1 complexed with camptothecin-loaded micelles. After 2 weeks, tissues were taken from untreated rats (**a**) and rats treated with naked siRaf-1 (**b**), methoxy poly(ethyleneglycol)-poly(ε-caprolactone) (MPEG-PCL-Tat/siRaf-1 complex) (**c**), CPT-loaded MPEG-PCL-Tat/siControl (**d**), and CPT-loaded MPEG-PCL-Tat/siRaf-1 (**e**). Adapted from Molecular Pharmaceutics with permission from the American Chemical Society, published by the American Chemical Society 2014, Copyright 2014 [13].

Kadari et al. introduced Angiopep-2 into solid lipid nanoparticles (SLN) to deliver DTX (A-SLN) against U87MG human glioma [83]. The A-SLN demonstrated strong inhibition of cell proliferation after 24 h of treatment compared to controls, a percentage of apoptosis of 45.9% and observed the presence of apoptotic bodies. In vivo studies demonstrated an increased rate survival of about 39 days and no side effects associated with A-SLN treatment [83]; as chemotherapy causes severe side effects due to lack of selectivity. Another strategy proposes the utilization of other pharmaceutical alternatives, as small-interfering RNA (siRNA), that are capable of silencing the expression of the defined gene, that in cancer, could be crucial to cellular growth. Kanazawa et al. developed a system for intranasal delivery of siRNA loaded in methoxy-PEG (MPEG)-PCL)-TAT micelles [84]. The results demonstrated that this conjugation accelerated transport along olfactory and trigeminal nerves and enabled the delivery in the brain [84]. Subsequently, the construct was loaded with camptothecin to target glioblastoma through intranasal delivery. The authors demonstrated that non-invasive routes by intranasal administration were more efficient for drug delivery than intravenous infusion. Nose-to-brain delivery resulted in reduction of tumour size and prolonged survival time for 28 days compared to 16.6 days in control mice (Figure 5) [13]. This CPP/NP conjugation as a delivery system, enhanced circulation time, BBB permeability, improved glioma targeting and selectivity and anti-tumour activity. 

The idea of CPP/NP conjugation was translated to other types and different cancers in order to achieve better treatment and development of new alternatives. For instance, breast cancer affects over 2 million women in 2018 and is considered the most frequent cause of death among women worldwide. The current major challenge in breast cancer treatment is the ability to predict therapeutic resistance and how to select the ideal drug for each individual [85,86]. Therefore, Hossain et al. monitored time-dependent anticancer drug delivery kinetics through surface-enhanced Raman spectroscopy (SERS) [87]. For this purpose, they constructed a biohybrid nanoparticle composed of gold NP, TAT and breast cancer antibody (anti-HER2) and analyzed the release rate of doxubicin (DOX). SERS map images showed higher signals from the biohybrid constructed in SK-BR-3 cell line (breast cancer) when compared to SH-SY5Y (neuroblastoma), and DOX intracellular release rate was more pronounced up to 12h after treatment [87]. In another study, CPP TAT was conjugated with gold NP platform to carry DOX against brain metastatic breast cancer [88]. The authors demonstrated that DOX was able to accumulate in intracranial brain metastasis in vivo and continuous treatment resulted in an increase life-span of 21.9% compared to phosphate-buffered saline (PBS) treatment [88]. Furthermore, Farkhani et al. evaluated the effect of CPP RW incorporated in silver NP (AgNP) against MCF-7 cell-lines [18]. The results demonstrated that AgNP were able to reduce growth of MCF-7 in a dose dependent manner. In addition, RW conjugation was essential for a 10-fold increase in anti-tumour activity (AgNP-RW cytotoxicity at 10.2 μg/mL and AgNP at 104.1 μg/mL) [18]. 

This conjugation was further explored in skin cancer for topical administration due to the potential of CPPs to permeate into skin layers and deliver the desire compounds [89]. Patlolla et al. formulated skin permeation of CPPs coated NP into the skin and demonstrated that NP functionalized with TAT showed a 3-fold increase in skin penetration compared to NP alone to a depth of 120 µM [89]. In other work by Asai et al., siRNA molecules were incorporated into a CPP derived from protamine-decorated lipid NP (CPP-LNP-siRNA) against melanoma and fibrosarcoma cell-line [19]. The authors observed that CPP incorporation lead to a significant enhancement of the interaction with both cell-lines. In addition, through confocal microscopy the authors demonstrated the cytoplasmatic distribution of siRNAs inside the cells and efficient silencing of 70% with no toxicity associated [19]. 

In the area of hepatocarcinomas, some studies with CPP/NP conjugation have shown good results and nanocarriers have been widely developed in this field. Zhang’s work validated the capacity of TAT incorporation into poly PLGA NP to efficiently delivery epirubicin against hepatic cancer and neuroblastoma cell-lines [90]. The construct demonstrated a sustainable release of about 38.3% in 48 h and intravenous administration of 4 µg/mL resulted in inhibition of tumour growth after 15 days of treatment [90]. Jin et al. further modified their CPP/NP/drug conjugated with the addition of a target moiety—HAb18F(ab specific antibody—that interacts with HAb186 over expressed in hepatocarcinoma tumours [91]. The authors demonstrated PTX-loaded NP and polyarginine peptide conjugation was successful to target liver tumour cells and showed significant anticancer activity among all formulations tested. After 3 days of treatment, tumours growth was about 77.3% and mice presented a long life-span of 35.2 days compared to control [91]. Using a different approach, Kapur et al. decided to test gold NP (AuNP) as a toxic compound and made AuNP selective with the conjugation of SVS-1 CPP specially designed as an anticancer peptide to facilitate efficiently delivery. Results demonstrated that SVS-1 incorporation promoted intracellular delivery of Au NP through direct translocation [92].

Other studies have proposed the utilization of this CPP/NP strategy in the area of lung cancer. Gao et al. used a RLW CPP anchored to PEG-PCL NP (RNP) to target and deliver DTX in a lung cancer cell model (A549) [93]. The results of cellular uptake showed a 4.5-fold increase in apoptosis percentage in RNP complexes. In addition, the results of RNP-DTX treatment in A549 spheroid tumours resulted in enhanced core penetration when compared to periphery distribution by NP alone [93]. Furthermore, Zhu et al. used an ACPP cleavable by matrix metalloproteinase (MMP) that is over expressed in many malignant tumours to enhance selectivity of the conjugate [94]. The authors synthesized a self-assembly drug-polymer prodrug consisting of: MMP2 cleavable peptide, TAT protected with PEG and loaded with PTX. Interestingly, nano preparations accumulated in tumour sites and cleavable MMP2 peptide was broken, which allowed the exposure of TAT promoting cell internalization and drug release [94].

In a recent study, Carnevale et al. designed and tested CPP/NP conjugates against drug resistant cancer cell-lines. The first phase of this study demonstrated CPPs differences in cell selectivity attached to quantum dots (QD) in resistant cell lines versus naïve [95]. The authors observed that different CPPs exhibit selective uptake profiles for each cell-line. Although prominent uptake profiles were observed for the hCT (9–32), Ku-70 and HSV1-VP22 in resistant melanoma and rat glioblastoma cells [95].

### 3.2. Cell-Penetrating Peptides and Nanoparticles as Imaging Agents

The development of non-invasive techniques is fundamental for research in order to integrate imaging technology with cellular and molecular biology. These techniques make it possible to acquire real-time information, and measurements of physiological or pathological processes at cellular and molecular level. They are also crucial in clinical practice to provide increased patient compliance, early detection, accurate diagnosis, drug development and efficacy of treatments. The main characteristics required are high resolution, highly sensitive instruments and specific imaging agents. Currently there are five imaging technologies accessible such as, X-ray, computed tomography imaging, optical imaging, radionuclide imaging (PET and SPECT), ultrasound imaging (US) and magnetic resonance imaging (MRI). The success of these techniques is determined by the specificity and sensitivity of the imaging agents used [96]. 

**Figure 6:**
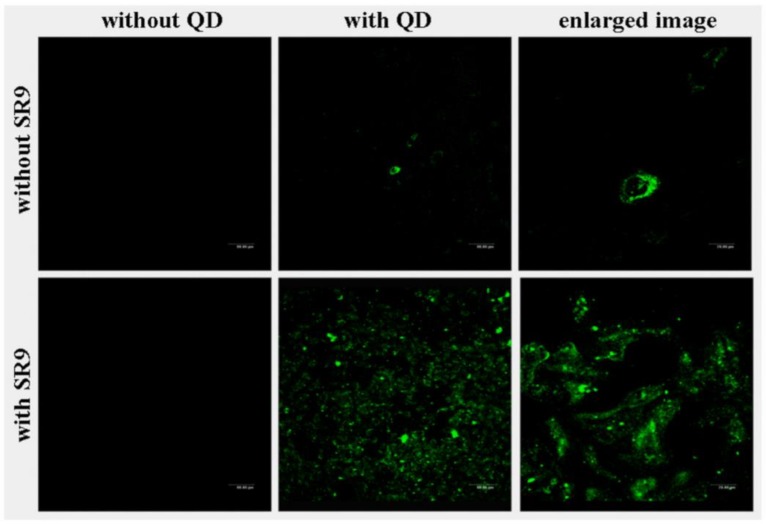
Confocal images of cellular uptake of CPP/quantum dots different combinations of free SR9, free quantum dots (QD) and SR9/QD complexes. Adapted from Journal of Nanoscience and Nanotechnology with permission from correspondent author Han-Jung Lee, published by HHS Public Access 2010, Copyright 2010 [97].

Chemical technology and nanotechnology have stimulated the development of new, selective imaging agents. The current research focus is the development of multifunctional molecular imaging agents to enhance the imaging abilities of the molecules, incorporate drugs and analyse the pharmacokinetics and pharmacodynamics of the therapy. Therefore, a variety of CPPs conjugated with imaging agents has been developed so far [52]. For example, in the work of Liu et al. paramagnetic particles linked to a CPPs to track mesenchymal stem cells through MRI were developed [21]. This work demonstrated that the developed gadolinium diethylenetriamine pentaacetic acid NP(Gd-DTPA-CPPs) were successfully detected inside mesenchymal stem cells in vitro by MRI [21]. Years later in 2010, Liu et al. were able to overcome the QD drawback of poor penetration in cells with an association with a CPP [97]. The work demonstrated that QD conjugated with arginine-rich CPP were taken up by living cells in vitro (Figure 6) [97]. 

In the same year, Li et al. team designed a multifunctional anti-tumour drug nanocarrier with the introduction of an activable enzyme with a CPP sequence TAT into mesoporous silica-coated QD for both DOX delivery against cancer and observations in real-time of the nuclear internalization [98]. The authors demonstrated the success of this complex in the nuclear delivery of DOX with associated anti-tumour activity against a variety of cancer cell lines, including resistant strains [98]. Current clinical cancer staging is determined by anatomical imaging such as X-ray, computer tomography, MRI and PET. Olson et al. modified an ACPP with the addition of a linker sensitive to proteases of tumour invasion and metastasis MMP2 and MMP-9 [10]. Further, Cys fluorescence label and polyamidoamine (PAMAM) dendrimer were added as the core of macromolecule. The work demonstrated higher fluorescence in tumours when ACPP was injected and also showed enhanced stability up to 48 h. In addition, the results demonstrated the efficiency of ACPP to mark both the breast tumour core and its respective lung metastasis. These results for MRI imaging further demonstrated the potential of this ACPP modified vectors for tumour delimitation and tracking in clinical usage [10]. 

Paramagnetic gadolinium and superparamagnetic iron oxide (SPIO) NP are the most widely used contrast agent because of low toxicity. Ding et al. demonstrated that incorporation of CPP in SPIO NP improved internalization into target cell and MRI sensitivity to tumours [99]. A time-dependent uptake range of 0–4 h of SPIO-R11 was found, and no toxicity was reported [99]. In a recent paper, Qian et al. proposed an interesting idea to take advantage of two tumour characteristics including low pH and angiogenesis receptors overexpressed (VEGFR2) and developed a nano construct with two CPPs and an AIE system to evaluate anticancer activity with apoptosis monitoring and delivery capability of the drug [100]. In this study, the results demonstrated that smart nano delivery efficiently accumulated in tumour sites and induced apoptosis in treated cells [100]. Moreover, Yong et al. investigated the use of permeation enhancers based on organic solvents [101]. They demonstrated that the addition of organic solvents, such as dimethyl sulfoxide, to the aqueous nanomaterial solution can enhance intracellular vesicle escape without compromising membrane integrity. Through conjugation of QD NP with CPP and organic solvent, the authors observed a significant enhancement in translocation across cell membranes [101].

### 3.3. Cell-Penetrating Peptides and Nanoparticles in Inflammation

Inflammation is considered as an adaptive response to restore homeostasis and is triggered by a stimulus or a condition. Inflammation responses occur in a wide variety of chronic cutaneous, allergenic and rheumatologic diseases and infections, which contribute to exacerbation of tissue damage and pain [102,103]. The mechanisms of chronic inflammation in case of autoimmune disease, chronic infections, diabetes type 2 and cardiovascular disease are still poorly understood and need thorough investigation [104]. A group of researchers developed a specialized thermosensitive polymer poly(*N*-isopylacrylamide) (poly(NIPAm) nanoparticles capable of establishing enhanced electrostatic interactions with CPP. In addition, there has been development of the CPP KAFAK characterized to target and inhibit mitogen activated protein kinase 2 (MK2) involved in the expression pro-inflammatory cytokines [74]. Conjugation of polyNIPAm-*co*-KAFAK suppressed TNF-alfa expression, thus inhibiting the pro-inflammatory cytokine cascade in macrophages. Moreover, they also tested the compound potential in ex vivo experiments and obtained similar inhibition effects and selectivity on damaged tissue, compared to healthy tissue [74]. Later, another study by this team applied the same goal to decrease the expression of pro-inflammatory cytokines from macrophages with KAFAK, upon conjugation with biocompatible thermosensitive polymer polyNIPAm and PEG. Enhanced release of KAFAK in the interior of the RAW-264.7 was found, inhibiting the secretion of pro-inflammatory cytokines. The work of Kim et al. focused on psoriasis that usually has STAT3 constitutively activated promoting disease expression and exacerbation [105]. The authors developed a CPP that specifically binds to STAT3 called APTstat3 tagged with 9-arginine CPP and conjugated with discoid-shaped lipid NP ((APTstat3-9R)-DLNP) in order to potentiate transcutaneous delivery against psoriasis. The incorporation of the NP increased penetration into the skin when compared with APTstat-9R alone. Furthermore, (APTstat3-9R)-DLNP complex effectively reduced psoriasis-like skin inflammation in mice ears with decreases of pathological findings in histologic observations [105]. Rheumatoid arthritis is a systemic chronic inflammatory disease that affects 1% of the adult population and is characterized by progressive joint destruction. One crucial limitation is that continuous treatment with methotrexate (MTX) leads to the development of drug resistance [106]. Zhao et al. developed a lipid polymeric hybrid nanoparticle (LNP) conjugated with stearic acid-octarginine, folate-PEG-PLGA and PK3 to deliver MTX [107]. The authors used folate to promote specific binding to selective receptors on the surface of activated macrophages, and hydrophobic core PLGA/PK3 served as a pH sensitive switch. Incorporation of Sta-R8 and folate increased the uptake efficiency and cytotoxicity in inflammatory sites. In vivo results showed therapeutic effects increased for adjuvant-induced arthritis in rats [107]. 

### 3.4. Cell-Penetrating Peptides and Nanoparticles in Central Nervous System Disorders

Currently, there is no cure for neurodegenerative diseases and current therapeutics were developed to address symptoms and not the mechanisms underlying the disease. Kesselheim’s work demonstrated that the development of CNS drugs has slowed down since 1990 for both early and late stages of clinical trials. So it is important to refocus attention on these diseases and develop new, effective compounds [108]. The CPP-derived peptide therapeutics were also designed and developed for the treatment of neurodegenerative disorders [109]. Insulin has an important role in neurodegerative diseases such as dementia, autism and Alzheimer’s and is responsible for improving cognitive function, learning and memory. Yan et al. developed a Tat mediated PLGA nanoparticle in order to deliver insulin to the brain via a non-invasive pathway (nose-to-brain route) [110]. This approach resulted in a 4.5-fold increase in intracellular accumulation of insulin [110]. Another work of Huang et al. used the same Angiopept-2/nanoparticle strategy but studied its potential to deliver a therapeutic gene in Parkinson disease models [111]. The authors prepared Angiopep-2 conjugated with dendrigraft poly-l-Lysine (DGL) PEG and concluded that long term administration of the compound improved locomotor activity and recovery of dopaminergic neurons in behavioural studies [111]. Another study demonstrated the importance of K16ApoE CPP incorporation in NP. The CPP K16ApoE improved permeability across BBB and brain accumulation of the construct up to 8–10 times higher than formulations without CPP [112].

## 4. Conclusions

Overall, the research that has been reported in the literature clearly indicates CPPs as a potential therapeutic tool against cancer, inflammation, cardiovascular disease and CNS disorders. However, some more specific studies on characterization, mode of action and performance must be carried out to achieve more successful treatments. The extraordinary breakthroughs in the development of CPPs have resulted in more stable and resistant complexes, enhanced efficacy and selective targeting. One of these is the development of dual delivery systems combining the positive properties of CPPs and NP, resulting in enhanced performance, precise delivery, increased half-time life, stability and higher drug loading. 

In conclusion, these recent advances and innovations have revealed that CPP conjugation with NP presents outstanding therapeutic delivery potential as non-toxic compounds that can be applied in the near future for treating different diseases, including so far, unmet medical needs.

## Figures and Tables

**Table 1:** Molecular structure of some cell-penetrating peptides (CPPs) referred to in the present review.

CPPs	Structure/Sequence	Ref.
**TAT**	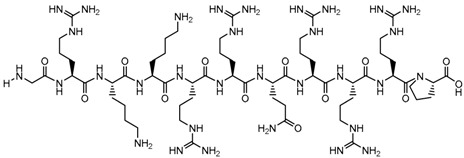 GRKKRRQRRRP	[4]
**R8**	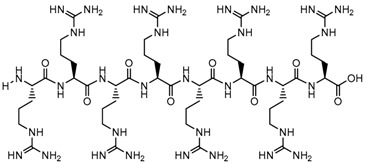 RRRRRRRR	[15]
**TGN**	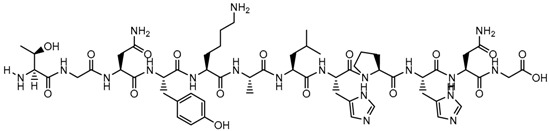 TGNYKALHPHNG	[16]
**Derived Ku-70**	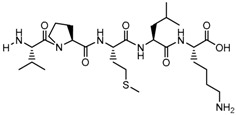 VPMLK	[17]
**RW(n)**	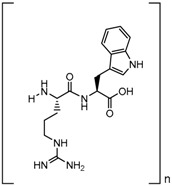 RW(*n*)	[18]
**CPP (RRRRRRGGRRRRG)**	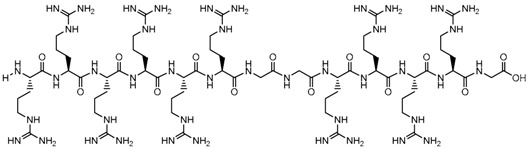 RRRRRRGGRRRRG	[19]
**SVS-1**	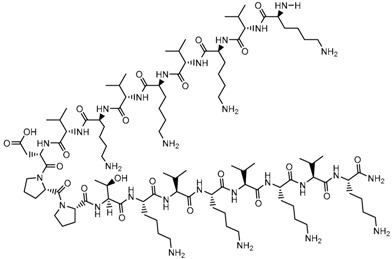 KVKVKVKVDPPTKVKVKVK-NH2	[20]
**L-CPP**	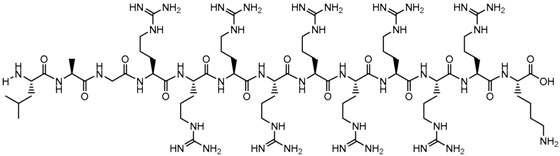 LAGRRRRRRRRRK	[21]
**RLW**	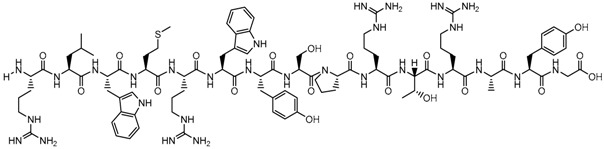 RLWMRWYSPRTRAYG	[22]
**K16ApoE**	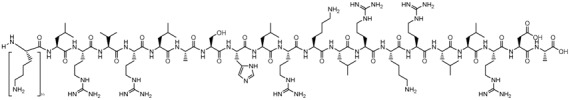 KKKKKKKKKKKKKKKKLRVRLASHLRKLRKRLLRDA	[23]
**Angiopep-2**	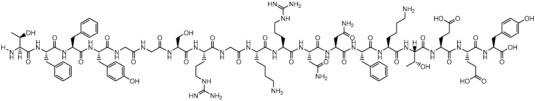 TFFYGGSRGKRNNFKTEEY	[24]
**ACPP**	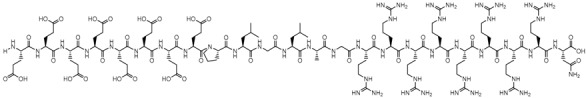 EEEEEEEEPLGLAGRRRRRRRRN	[12]
**KAFAK**	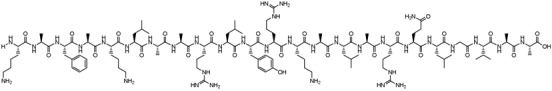 KAFAKLAARLYRKALARQLGVAA	[25]
**hCT (9–32)**	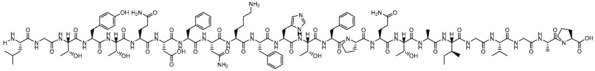 LGTYTQDFNKFHTFPQTAIGVGAP	[26]
**VP22**	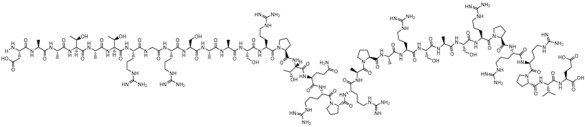 DAATATRGRSAASRPTQRPRAPARSASRPRRPVE	[27]

**Table 2:** Advantages and disadvantages of CPP conjugation with NP.

CPP Conjugation with NP	
Advantages	Disadvantages
Selective targeting Intracellular delivery Delivery of therapeutic compounds Low toxicity Bioaction High Stability	Resistance to drug Drug release Aggregation Endosomal entrapment

**Table 3:** Conjugation strategy and different applications.

Construct	CPP	NP	Delivery Cargo	Applications	Ref.
MPEG-PCL-TAT+siRNA micelles	TAT	Methoxypoly(ethylene glycol) and poly(ε-caprolactone)	siRNA	Brain cancer	[84]
TGN-NP	TGN	PEG-PLGA	Coumarin-6	BBB targeting	[16]
MPEG-PCL-TAT micelles	TAT	MPEG-PCL	Anti RafsiRNA/CPT drug	Glioblastoma	[13]
ANG/TAT/DTX	Angiopep-2 and TAT	Tandem micelles	DTX	Glioblastoma	[82]
AsTNP	TGN	PEG-PCL	DTX	Glioblastoma	[77]
ANG-NP-PTX	Angiopep-2	PEG-PCL	PTX	Glioblastoma	[79]
A-SLN	Angiopep-2	Solid lipid nanoparticle	DTX/DiR/coumarin-6	Glioblastoma	[83]
AnACNPs	ACPP and Angiopep-2	PEG-PCL	Coumarin-6/DiR	Glioblastoma	[80]
AnACNps	ACPP and Angiopep-2	PEG-PCL NP	DTX	Glioblastoma	[81]
CPP-QD@mSiO_2_	ACPP	Mesoporous silica-coated QD	DOX	Cancer	[98]
TAT-Au-DOX	TAT	PEG and dodecylamine coated Au NP	DOX	Breast metastatic cancer	[88]
AuNP/DOX/TAT/PEG/antibody	TAT-C	Au NP	DOX	Breast cancer	[87]
AgNP-RWn	[RW]3,4,5,6	Silver NP (AgNP)	----	Breast cancer	[18]
PLGA-TAT-ADM	TAT	Poly(lactic-glycolic acid)	Epirubicin	Hepatic cancer	[90]
H+C-NPs	R9	PLGA-PEG	PTX	Hepatic cancer	[91]
TNPs	RLW	PEG-PCL	DTX/coumarin-6/DiR	Lung cancer	[93]
PEG2000-peptide-PTX and TATp-PEG1000-PE	TAT	PEG1000-PE	PTX	Lung cancer	[94]
CPP-LNP-siRNA	Arginine rich and siRNA carrier	Protamine-decorated lipid NP	SiRNA GFP/luciferase	Melanoma cancer	[19]
AuNP-SVS-1/TXR	SVS-1	AuNP	Texas red dye (TXR)	Cancer	[92]
QD-CPP	Ku-70; TAT; hCT; HSV1-VP22;	Quantum dot (CdTE@CdSe@CdS@ZnS)	----	Multidrug resistant cancer	[95]
STD-NMdrug	TAT	Nanomicelles	DOX	Cancer and Imaging agent	[100]
QD-TAT	TAT	Quantum dots coated PEG	----	Imaging agent	[101]
SPIO-R11	Arginine rich peptide (R11)	Supermagnetic iron oxide (SPIO)	----	Imaging agent MRI (Bladder cancer)	[99]
Gd-DTPA-CPP	L-CPP	DTPA	Gadolinium	Imaging agent	[21]
SR9-QD	R9	Quantum dots (CdSe/Z S) core shell	----	Imaging agent	[97]
ACPP-GD	ACPP	PAMAM dendrimer	Gadolinium	Imaging agent	[10]
Poli(NIPAM)-KAFAK	KAFAK	Thermosensitive polymer poly(*N*-isopropylacrylamine)	KAFAK	Inflammation	[74]
Sta-R8+FA- PPLPNs/MTX	R8	Lipid polymeric hybrid NP	MTX	Rheumatoid arthritis	[107]
APTstat3-9R	R9	Discoid shaped lipid NP	STAT	Inflammation	[105]
K16APoE-TargetedNP-DutchAβ40	K16ApoE	PGLA	Curcumin	Alzheimer model	[112]
Insulin-loaded-TAT-NP	TAT	PLGA	Insulin	Alzheimer disease	[110]
DPA-hGDNF	Angiopep-2	Dendigraft poly-l-lysine (DGL-PEG) and PAMAM dendrimer	Gene therapy (hGDNF)	Parkinson disease	[111]
MPEG-PCL-TAT+siRNA micelles	TAT	Poly(ethylene glycol) and poly(ε-caprolactone)	siRNA	Brain cancer	[84]

Cell-penetrating peptides (CPP); Quantum dot (QD); Supermagnectic iron oxide (SPIO); Poly(ethylene glycol) (PEG); poly(ε-caprolactone) (PCL); nanoparticles (NP); Silver (Ag); gold (Au); Solid Lipid Nanoparticle (SLN); Poly(lactic-glycolic acid) (PLGA); polyamidoamine (PAMAM); Methotrexate (MTX); discoid-shaped lipid NP (DLNP); diethylenetriamine pentaacetic acid (DTPA); placlitaxel (PTX); Doxubicin (DOX); Docetaxel (DTX); small-interfering RNA (SiRNA); Green Fluorescence protein (GFP); Thermosensitive polymer poly(*N*-isopropylacrylamine) (poli(NIPAM); Sendigraft poly-l-Lysine (DGL); Blood–brain barrier (BBB); Magnetic resonance imaging (MRI).
